# Clinical, Genetic, and Protein Structural Aspects of Familial Dysalbuminemic Hyperthyroxinemia and Hypertriiodothyroninemia

**DOI:** 10.3389/fendo.2017.00297

**Published:** 2017-11-01

**Authors:** Ulrich Kragh-Hansen, Monica Galliano, Lorenzo Minchiotti

**Affiliations:** ^1^Department of Biomedicine, University of Aarhus, Aarhus, Denmark; ^2^Department of Molecular Medicine, University of Pavia, Pavia, Italy

**Keywords:** l-thyroxine, euthyroid hyperthyroxinemia, triiodothyronine, albumin, prevalence, binding sites, mutations

## Abstract

Familial dysalbuminemic hyperthyroxinemia (FDH-T4) and hypertriiodothyroninemia (FDH-T3) are dominantly inherited syndromes characterized by a high concentration of thyroid hormone in the blood stream. The syndromes do not cause disease, because the concentration of free hormone is normal, but affected individuals are at risk of erroneous treatment. FDH-T4 is the most common cause of euthyroid hyperthyroxinemia in Caucasian populations in which its prevalence is about 1 in 10,000 individuals, but the prevalence can be much higher in some ethnic groups. The condition is caused by a genetic variant of human serum albumin (HSA); Arg218 is mutated to histidine, proline, or serine or Arg222 is changed to isoleucine. The disorder is characterized by greater elevation in serum l-thyroxine (T4) than in serum triiodothyronine (T3); T4 can be increased by a factor 8–15. The high serum concentration of T4 is due to modification of a binding site located in the N-terminal half of HSA (in subdomain IIA). Thus, mutating Arg218 or Arg222 for a smaller amino acid reduces the steric restrictions in the site and creates a high-affinity binding site. The mutations can also affect binding of other ligands and can perhaps cause modified pharmacokinetics of albumin-binding drugs. In normal HSA, the high-affinity site has another location (in subdomain IIIB). Different locations of these sites imply that persons with and without FDH-T4 can have different types of interactions, and thereby complications, when given albumin-binding drugs. FDH-T3 is caused by a leucine to proline mutation in position 66 of HSA, which results in a large increment of the binding affinity for T3 but not for T4. For avoiding unwanted treatment of euthyroid persons with hyperthyroxinemia or hypertriiodothyroninemia, protein sequencing and/or sequencing of the albumin gene should be performed.

## Introduction

**Figure 1:**
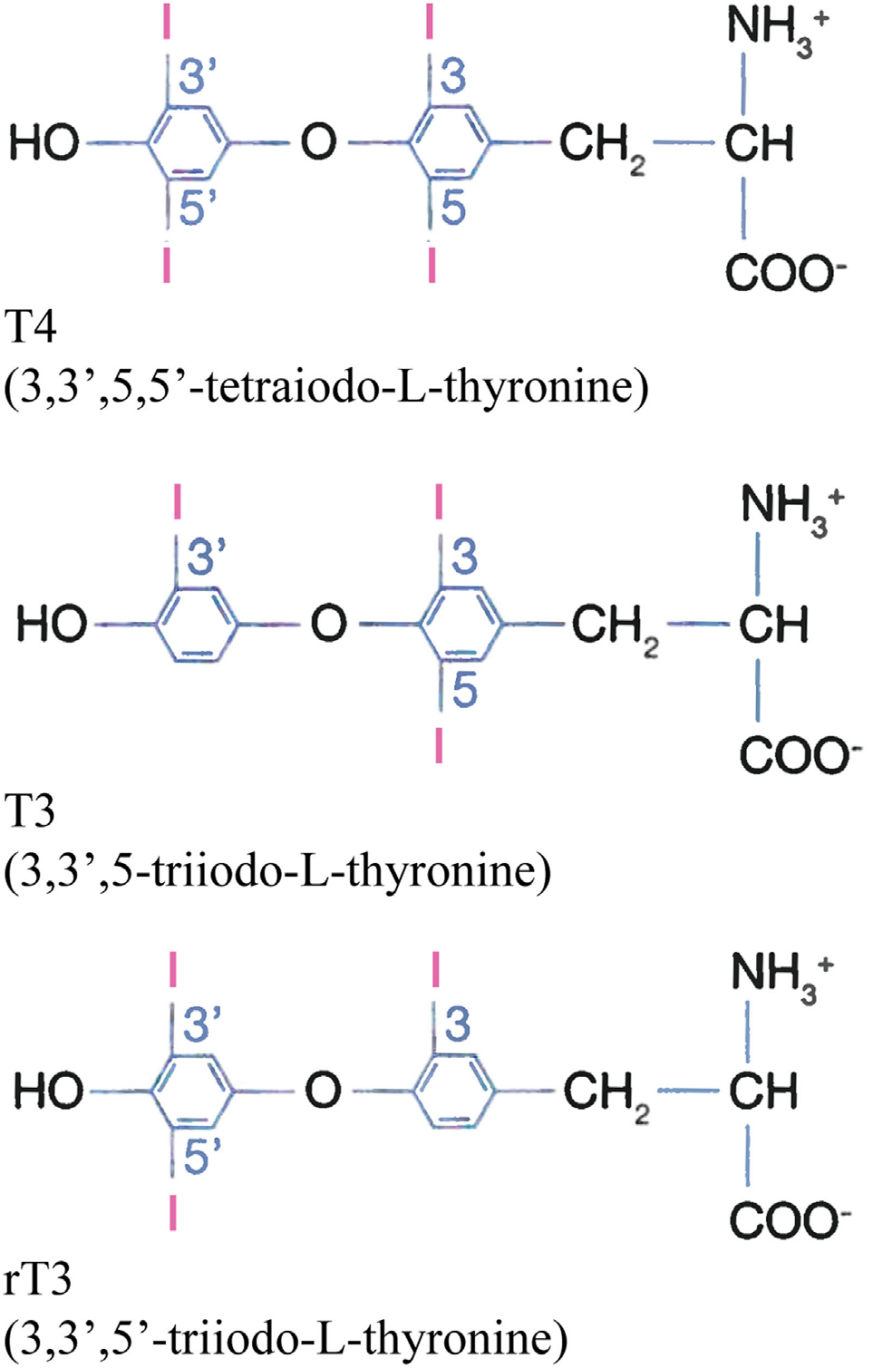
Chemical structures of T4, T3, and the biologically inactive rT3 at physiological pH.

The hormones l-thyroxine (T4) and its active form, i.e., triiodothyronine (T3) (Figure 1), act on almost all cells in the human body and are necessary for normal physical and mental development. In addition, they are very important for maintaining the basal metabolic rate. T4 is secreted into the bloodstream by the thyroid gland. The gland also secretes some T3 but the major part of T3 is generated by deiodination of T4 in peripheral tissues. T4 and T3 are hydrophobic molecules and their solubility in aqueous media is relatively low. However, their concentrations in humans are much increased through reversible binding to proteins, which are distributed throughout the body. The most important of these proteins are thyroxine-binding globulin (TBG), transthyretin (TTR), and human serum albumin (HSA), and 99.97 and 99.7% of extracellular T4 and T3, respectively, are protein-bound (1). Binding also results in increased circulation time of the hormones, because TBG, TTR, and HSA have half-lives of 5 (2), 2 (2), and 19 (3) days, respectively. Finally, the proteins serve as circulating depots and transporters for the hormones, maintaining a large extrathyroidal reserve of T4 and T3, and avoid excessive urinary iodine loss (1).

The mature TBG is a single polypeptide chain of about 54 kDa consisting of 395 amino acids with four N-linked oligosaccharides (1). Its concentration in serum is only 16 mg/L (0.3 µM) but due to high-binding constants for T4 (1 × 10^10^ M^−1^) and T3 (1 × 10^9^ M^−1^), the protein carries ca. 75% of both T4 and T3 in serum (1). TTR, previously known as thyroxine-binding prealbumin, is a non-glycosylated 55 kDa protein, consisting of four identical 127 amino acids subunits (1). TTR has a concentration of 250 mg/L (4.5 µM) and possesses two sites with primary binding constants of 2 × 10^8^ M^−1^ (T4) and 1 × 10^6^ M^−1^ (T3), and it binds 15–20% of T4 and ca. 5% of T3 in serum (1). Among the three proteins, HSA is found in the highest concentration, namely 42 g/L (0.6 mM) (3). The protein has more binding sites for the hormones but with the lowest high-affinity constants, i.e., in the order of 10^5^–10^6^ M^−1^ (1, 4), which results in binding of ca. 5% of T4 and ca. 20% of T3 (1). Thus, TBG binds most of the hormones under physiological conditions. However, HSA is the protein with the longest life span, and it provides an important fast-response reservoir for the hormones during capillary transit (5, 6).

Lipoproteins bind T4 with an affinity similar to that of TTR (7), but they only transport ca. 3% of total T4 (7, 8) and up to 6% of total T3 in serum (7). The relative distribution of T4 bound to the lipoproteins has been reported to be 0.008 [very low-density lipoproteins (VLDL)], 0.067 [low-density lipoproteins (LDL)], and 0.92 [high-density lipoproteins (HDL)] (8). In addition to carrying T4, LDL, but not TBG, TTR, or HSA, facilitates cellular uptake of the hormone *via* the LDL receptor (8, 9). HDL also increases cellular uptake of T4, but this effect is most probably due to increased facilitated diffusion through the cell membrane (9).

**Figure 2:**
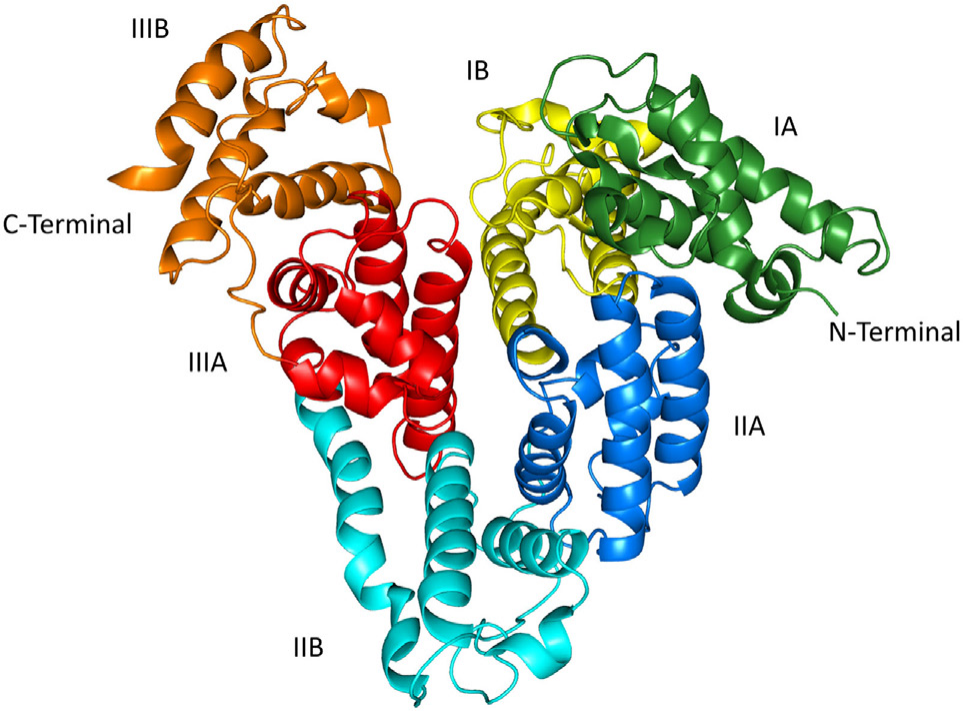
The structure of human serum albumin as revealed by X-ray crystallography (10, 11). The subdivision of the protein into domains (I–III) and subdomains **(A,B)** is indicated. The illustration was made with PyMol on the basis of the atomic coordinates (PDB ID: 1uor) available at the RCSB Protein Data Bank.

Human serum albumin is a single-chain protein synthesized in and continuously secreted from liver cells. Normally, it is a simple protein, i.e., it is without prosthetic groups and covalently bound carbohydrate or lipid (3). The polypeptide chain is composed of 585 amino acids, it has a molecular mass of 66.5 kDa and forms a heart-shaped structure (Figure 2) (10, 11). The protein has about 67% α-helix but no β-sheet and can be divided into three homologous domains (I-III). Each of these is composed of two subdomains (A and B). All but one, Cys34, of the 35 cysteine residues are involved in the formation of 17 stabilizing disulfide bonds. The total amount of HSA in the body is ca. 360 g, of which about two-thirds is outside the bloodstream and about one-third is in the bloodstream. However, the concentration of HSA is higher in the bloodstream, and that is why the protein can contribute with ca. 80% of the colloid osmotic pressure of plasma (12). In addition, HSA is regarded as the quantitatively most important circulating antioxidant, and it has enzymatic properties, which are so pronounced that they most probably are of biological importance (12).

The most well-studied function of HSA undoubtedly is its ability to reversible bind numerous endogenous and exogenous inorganic ions and organic molecules (3, 12). In the circulation, this function can be affected in different ways; binding of a ligand can be influenced by co-binding of another ligand, or binding can be altered by covalent modification of the protein as a result of, e.g., oxidation or glycation. The function can also be changed by genetic modification of the protein. In the case of especially T4, but also of T3, several genetic variants show altered binding, the most well-known of these are mutation of Arg218 or Arg222 in the case of T4 resulting in familial dysalbuminemic hyperthyroxinemia (FDH-T4) (OMIM #615999) and Lys66 in the case of T3 leading to familial dysalbuminemic hypertriiodothyroninemia (FDH-T3) (4). All the FDH-T4 and FDH-T3 patients so far described are heterozygous for the causative mutation.

Individuals with FDH-T4 or FDH-T3 present with altered thyroid function tests, but they are clinically euthyroid. Therefore, early identification of the syndromes is important to avoid unnecessary medical or surgical treatment. In the following, methods to detect FDH-T4 and FDH-T3, and factors which can influence detection, will be presented. Other conditions resulting in discordant thyroid function tests are mentioned. The most relevant clinical findings of published cases, ethnicity, countries of detection, and nucleotide and amino acid changes, will be tabulated and discussed. The structure of binding sites in native and mutated albumins are presented and analyzed based on surveys performed on genetic and recombinant variants as well as crystallographic and docking studies. Finally, the effect of the mutations on binding of other ligands and their potential clinical importance will be discussed.

## Diagnosis of and Differential Diagnosis to FDH-T4

Persons with FDH-T4 are usually characterized by having an elevated serum concentration of total T4 but a concentration of unbound T4 within the normal range, normal T4 production rate, and serum sex hormone-binding globulin concentration, and, finally, a normal TSH response to TRH (13). Several assays, however, result in falsely increased free T4, especially when an indirect or analog assay has been used (1, 2). In addition, reagent composition can affect the outcome of some assays. Instead, isoelectric focusing or immunoprecipitation (1), and more direct methods such as equilibrium dialysis or ultrafiltration (2) are recommended. In all cases, the syndrome should be further confirmed by family studies or sequencing of HSA itself or of its gene. Recently, a whole protein electrospray time-of-flight mass spectrometry method, combined with DNA sequencing, was developed. The test is rapid (<10 min) and requires only a minimal amount (<2 µL) of serum (14).

Structural changes of HSA can thus affect the total serum concentration of T4. By contrast, the hormone concentration is apparently not influenced by changes in albumin concentration. For example, total T4 seems to be unaffected by the complete or near complete absence of HSA, i.e., in analbuminemia (1, 2, 13). The latter is a rare congenital condition caused by different defects in the HSA gene (15, 16).

Abnormal thyroid function tests in euthyroid persons can also be caused by other means than FDH-T4. For example, the TTR variant A109T results in principally the same test results as FDH-T4, namely increased total T4 without proportional elevation in total T3 and non-suppressed serum TSH (13). In addition, interferences in thyroid hormone immunoassays by circulating endogenous antibodies such as thyroid hormone autoantibodies and human anti-mouse antibodies as well as rheumatoid factors are well known (17, 18). The presence of these factors may cause either falsely depressed or falsely increased values of thyroid hormones, depending on the nature of the interfering factor and the assay design (18). The prevalence of thyroid hormone antibodies has been reported to be 0–25% (18). The wide variation of prevalence could reflect differences in patient subgroups studied as well as differences in the detection methods used such as assay sensitivity and specificity (17, 18). However, in these cases, also the measured levels of the free fractions are too high (17). Furthermore, assays are affected by drugs such as amiodarone, which can inhibit the conversion of T4 to T3 (19). In addition, the concentrations of free T4 and T3 and perhaps also of TSH can be elevated in acute psychiatric disorders; the exact explanation for these effects is not known in detail but they seem to be multiplex (19). Differential diagnoses also include the sick euthyroid syndrome and thyrotropinoma. Finally, euthyroid individuals can show with increased serum hormone levels due to resistance to thyroid hormone mainly caused by mutations in the nuclear thyroid hormone receptor beta. In such cases, the increased serum hormone levels are due to reduced T3 binding to the receptor (20–23).

In certain diseases, the prevalence of thyroid hormone antibodies is much higher than 0–25%. For example, the prevalence is ca. 50% in primary Sjögren syndrome (24, 25), 92.3% in type 1 diabetes mellitus (25), and as high as 97% in vitiligo, which is strongly associated with autoimmune thyroid disorders (24).

Finally, it should be noted that FDH-T4, which does not call for treatment, could mask the simultaneous presence of true thyroid disease such as hypothyroidism, autoimmune thyroiditis, or thyrotoxicosis (1, 2, 26, 27).

## FDH-T4 Can Result in Inappropriate Treatment

The presence of FDH-T4 may be misdiagnosed as a thyroid disease state like thyrotoxicosis, and these persons may be subject to inappropriate diagnostic procedures and surgical or drug therapy. Thus, several cases of subtotal thyroidectomy and/or ^131^I-therapy have been reported. In addition, patients have been given antithyroid medication, e.g., carbimazole. A case from Denmark can illustrate how far-reaching effects an inappropriate treatment can have. A woman with FDH-T4 was erroneously given a high dose of thiamazole (40 mg/day). She became pregnant and because of fear for teratogenic effects caused by the medicine she was advised, and accepted, twice to get an induced abortion (28). Therefore, as always, “treat the patient, not the laboratory report.”

## FDH-T4 Causing Mutations

Single point mutations in exon 7 of the HSA gene (*ALB*) can change arginine in position 218 or 222 in the mature protein for another amino acid. Usually, these genetic variants have an increased affinity for T4. In the following, an overview is given of some of the genetic, protein structural, clinical, and prevalence aspects of this autosomal dominantly inherited condition.

### R218H

The molecular basis for this albumin variant is a guanine (*G*) to adenine (*A*) mutation in the second nucleotide of the codon for Arg218; thus, *CGC* has been changed to *CAC*. Often, this mutation is linked to the SacI^+^ polymorphism in the *ALB*. R218H was the first type of FDH-T4 reported, and it is the most common mutation that produces the syndrome. As seen in Table 1, so far, 67 cases in 24 families have been reported. To these numbers can be added some of the cases with more than one diagnosis referred to in Section “Diagnosis of and Differential Diagnosis to FDH-T4.” Thus, three members of a family have both FDH-T4 and congenital hypothyroidism (26). In another family, a young girl has autoimmune thyroid disease as well as FDH-T4, whereas her father has only FDH-T4 (27); he is included in Table 1. Geographically, this type of FDH-T4 has been detected in North America, Western Europe, Eastern Asia, and in New Zealand. By contrast, Japanese and Africans, for example, have not been reported to have this mutation. A founder effect for the R218H substitution is convincing to explain the appearance of this FDH-T4 form in some kindreds (29–31), but not in all the reported cases, because this mutation is located in a *CpG* dinucleotide hot spot, where the molecular defects are more likely to occur independently (see Apparent Hot Spots in the Albumin Gene).

**Table 1:** Molecular, clinical, and ethnic characteristics of familial dysalbuminemic hyperthyroxinemia (FDH-T4) (1–20) and FDH-T3 (21) causing mutations.

No.	Mutation	Base change^a^	Total T4 (μg/dL)^b^	Total T3 (ng/dL)^c^	Total rT3 (ng/dL)^d^	Persons (families)	Country	Ethnicity	Reference
1	R218H	c.725*G*>*A*	13.3–21.5	103–218	21.3–44.2	21 (8)	USA	Mainly European	(29)
2	R218H	c.725*G*>*A*	NI^e^	NI	NI	3 (3)	HI, USA	Caucasian	(32)
3	R218H	c.725*G*>*A*	15.4	147	28.6	22 (1)	USA	Amish (Swiss)	(30)
4	R218H	c.725*G*>*A*	15.4–18.8	130–150	NI	1 (1)	Taiwan	Chinese	(33)
5	R218H	c.725*G*>*A*	15.6	138	26.9	2 (1)	Puerto Rico	Hispanic	(31)
6	R218H	c.725*G*>*A*	14.9–20.0	NI	NI	7 (1)	Hong Kong	Chinese	(34)
7	R218H	c.725*G*>*A*	18.5	NI	NI	1 (1)	Denmark	Danish	(28)
8	R218H	c.725*G*>*A*	NI	NI	NI	4 (4)	Western Europe	NI	(35)
9	R218H	c.725*G*>*A*	13.7	119	43.6	1 (1)	USA	NI	(27)
10	R218H	c.725*G*>*A*	14.6	NI	NI	2 (2)	New Zealand/Sri Lanka	Caucasian/NI	(14)
11	R218H	c.725*G*>*A*	14.5	99	NI	3 (1)	Korea	Korean	(36)
12	R218P	c.725*G*>*C*	182	225	164	6 (1)	Japan	Japanese	(37)
13	R218P	c.725*G*>*C*	NI	NI	NI	2 (2)	Japan	Japanese	(38)
14	R218P	c.725*G*>*C*	102–120	214–312	156–177	4 (1)	Switzer-land	Swiss	(39)
15	R218P	c.725*G*>*C*	99.1	338	NI	1 (1)	Japan	Japanese	(40)
16	R218P	c.725*G*>*C*	>30	387	NI	3 (1)	Japan	Japanese	(41)
17	R218P	c.725*G*>*C*	24.9^f^	232	NI	4 (3)	Japan	Japanese	(42)
18	R218P	c.725*G*>*C*	>24.9	NI	NI	1 (1)	Japan	Japanese	(43)
19	R218S	c.724*C*>*A*	85	288	86.2	2 (1)	Canada	Bangla-deshi	(44)
20	R222I	c.737*G*>*T*	15.9-23.5	–^g^	–^g^	9 (4)	UK	Somali/Croatian	(45)
21	L66P	c.269*T*>*C*	8.4	256	NI	8 (1)	Thailand	Thai	(46)

*The hormone concentrations are usually given for the probands*.

*^a^Codon numbering according to HGVS rules and based on the cDNA sequence NM_000477.12*.

*^b^Normal concentration: 4.5–12 µg/dL (55–144 nmol/L)*.

*^c^Normal concentration: 90–180 ng/dL (0.9–2.8 nmol/L)*.

*^d^Normal concentration: 15–32 ng/dL (0.2–0.5 nmol/L)*.

*^e^No information available*.

*^f^Uncharacteristic low value determined 10 months after admission (42)*.

^g^See Section “R222I.”

The thyroid function tests show increased total T4 (but normal free T4) and, as apparent from Table 1, normal to slightly increased T3 and reverse T3 (rT3; Figure 1). Total T4 is only moderately increased; as compared to the upper limit of the normal concentration range it has been increased by a factor of 1.1–1.8 (Table 2).

**Table 2:** Summary of mutations and phenotypes.

Mutation	Factors by which hormone concentrations are increased^a^		
	Total T4	Total T3	Total rT3
R218H	1.1–1.8	0.6–1.2	0.7–1.4
R218P	8–15	1.2–2.1	5
R218S	7	1.6	2.4
R218C^b^	–	–	–
R222I	1.3–2.0	NI^c^	40–70
L66P	0.7	1.4	NI
L66V^b^	–	–	–

*^a^Concentrations are related to the upper limit of the normal concentration range*.

*^b^These variants are included in the Exome Aggregation Consortium Website (47), but they have not been reported to case FDH-T4 or FDH-T3*.

*^c^No information available*.

### R218P

This type of FDH-T4 is due to another missense mutation (*G* to *C*) of the same nucleotide, which results in replacement of the normal Arg218 with a proline. The mutation results in the presence of a restriction site for *Ava*II (40). R218P is the only FDH-T4 form found so far in Japan. Until now, it has been detected in 17 persons in 9 families (Table 1), most of them in Aomori prefecture (41). However, the mutation is not specific for Japan, because it has also been found in a Caucasian, Swiss family with no Asian ancestry (39).

The cases are characterized by having extremely high concentrations of total T4; the concentration is typically increased 8- to 15-fold (Table 2). In addition, total T3 and rT3 are elevated but to a lesser extent, i.e., by factors of 1.2–2.1 and ca. 5, respectively. Finally, usually also the concentration of free T4 and T3 are above normal. Dependent on the method used, free T4 and T3 can be increased 1.2–4.9 and 1.3–3.0 fold, respectively (37, 42, 43).

### R218S

This albumin variant is caused by a *C* to *A* mutation in the first nucleotide of the codon for Arg218; thus, *CGC* has been modified to *AGC*. At present, this albumin form has only been found in one family in Canada. The effect of the amino acid substitution on the serum concentrations of the thyroid hormones is intermediate to those typically found for R218H and R218P. Thus, total T4 is increased sevenfold, and total T3 and rT3 are elevated 1.6- and 2.4-fold, respectively (Table 2). As measured by equilibrium dialysis, free T4 was within the normal range (44).

### R218C

The Exome Aggregation Consortium Website (47) reports on the existence of a R218C mutant with a frequency of about 2.5 in 100,000. This albumin variant is caused by a missense mutation of the first nucleotide in the codon, i.e., *C* to *T*. To the best of our knowledge, the variant has not been reported as a cause of FDH-T4 nor has its T4 binding properties been studied *in vitro*. However, because cysteine is a smaller amino acid than arginine, it is possible that the isoform can result in FDH-T4; see “Structure and Location of T4-Binding Sites in HSA.”

### R222I

Mutation of the neighboring arginine residue can also result in FDH-T4 (Table 1). This has been found to be the case, when the second nucleotide in the codon for Arg222 (*AGA*) is changed to a thymine (*ATA*) (45). So far, the mutation has been detected in one family of Caucasian East European (Croatian) and in three unrelated families of East African (Somali) origin. The latter examples seem to be the first ones of FDH-T4 among Africans.

Total T4 is increased by a factor of 1.3–2.0 (Table 2), and total T3 are slightly increased or normal (45). Free T4 was normal as measured by equilibrium dialysis but increased when determined by different one-step and two-step platforms. With respect to these parameters, the condition resembles that of R218H, see above. By contrast, in the case of R222I, the total concentration of rT3 is increased very much, i.e., 40- to 70-fold (45).

### Other Mutations

High-affinity binding of T4 to 32 other genetic variants of HSA has been examined by equilibrium dialysis, and the studies revealed two additional cases of increased binding and eight examples of decreased binding (4). Almost all of the modified bindings were caused by mutations in domain III, mainly in subdomain IIIB (Figure 2). Whether these altered affinities have clinical consequences is not known at present.

## Apparent Hot Spots in the Albumin Gene

The presence of hypermutable *CpG* sequences in *ALB* has previously been proposed to explain the relatively high frequency of some genetic variants of the protein (48, 49) or the presence of the same mutation in two unrelated analbuminaemic individuals (15). These mutations occur in cytosine residues in *CpG* sequences in either the sense or the antisense strand, giving rise to *CpG* to *TpG* or *CpA* transitions. A *G* to *A* transition in the second nucleotide of the codon for Arg218 (c.725*G*>*A*) in a *CpG* sequence is the molecular basis of the R218H mutation, which is by far the most common cause of FDH-T4. This mutation was found in many different countries and ethnicities (Table 1), thus suggesting that the *CpG* sequence in the codon for Arg218 represents a hot-spot in *ALB*. Also, the c.724*C*>*T* (R218C) mutation, present in the Exome Aggregation Consortium Website (47), is in this *CpG* sequence, but it has not so far been reported as a cause of FDH-T4.

## Prevalence

Familial dysalbuminemic hyperthyroxinemia is the most common cause of inherited euthyroid hyperthyroxinemia in the Caucasian population with an estimated prevalence of 1 in 10,000 individuals (36, 43, 45). However, the prevalence in subjects of Hispanic origin is much higher, i.e., 1.0–1.8% (14, 32, 43). In countries like Venezuela (0.17%), France (0.08%), and Denmark (0.01%), the prevalence is high (29), whereas the condition is extremely rare in Japan (43).

According to the Exome Aggregation Consortium Website (47), the allele frequency of R218H in the average population is about 7.4 in 100,000. By contrast, the frequencies of R218P, R218S, and R222I are very low; less than 0.83 in 100,000.

The prevalence of FDH-T4 is most probably underestimated and underdiagnosed owing to insufficient clinical suspicion. Clinicians should consider the possibility of FDH-T4 in subjects with a clinically euthyroid state but with abnormal results of thyroid function tests (36).

Less information seems to exist on the prevalence of genetic variants of TBG and, especially, TTR (1). Until this year, 49 variants of TBG have been found and genetically characterized. The most common type of these results in partial deficiency and has a prevalence of 1 in 4,000 newborns. Genetic defects can also cause complete TBG deficiency, and this syndrome appears with a prevalence of 1 in 15,000 individuals. On the other hand, TBG excess has an estimated prevalence of about 1:25,000 and is caused by gene duplication or triplication (1). So far, more than 70 TTR gene mutations have been identified; apparently with no known prevalence (1). Six of these have reduced binding affinity for T4, whereas 4 isoforms bind the hormone stronger. However, in all cases, the serum concentration of T4 (and T3) is only minimally affected, because TTR only binds a low amount of thyroid hormone.

## Structure and Location of T4-Binding Sites in HSA

**Figure 3:**
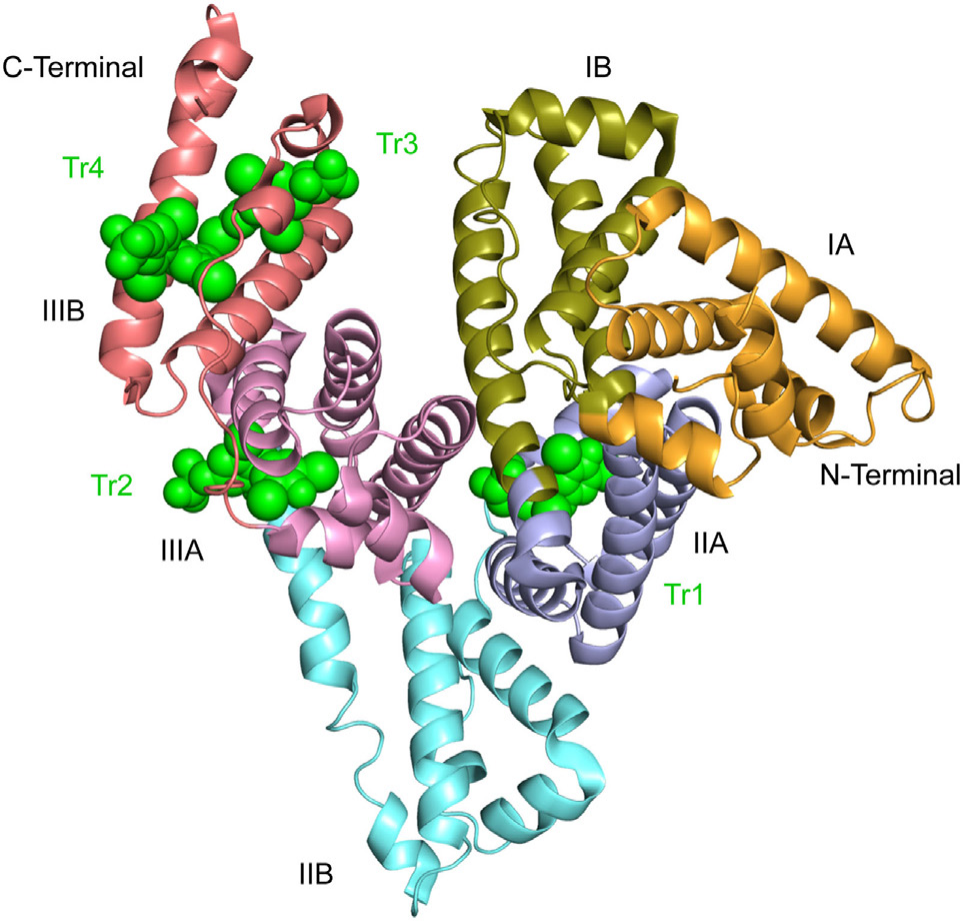
Crystal structure of defatted rHSA complexed with l-thyroxine. The structure suggests the existence of four binding sites numbered Tr1 to Tr4 from the N-terminal end. The hormone is depicted in space-filling representation and in green. The subdivision of the protein into domains (I–III) and subdomains **(A,B)** is indicated. This illustration and Figures 4–6 are 2.65 Å structures (6) and are made with PyMol on the basis of the atomic coordinates 1HK1 available at the RCSB Protein Data Bank.

Detailed information about the T4-binding sites has been obtained by approaches like X-ray crystallography, binding experiments with recombinant and genetic mutants and molecular docking methods. Thus, structural characterization of complexes formed by high concentrations of T4 and defatted, recombinant HSA (rHSA) proposes the existence of four hormone-binding sites numbered Tr1 to Tr4 from the N-terminus of the protein (PDB ID: 1HK1) (6) (Figure 3). Of these sites, the existence of site Tr3 could be questionable, because it is formed by two symmetry-related albumin molecules in the crystal (6). The structures of defatted, recombinant forms of the mutants R218H (PDB ID: 1HK2) and R218P (PDB ID: 1HK3) to which T4 has been bound are also known. Finally, the structure has been determined of ternary HSA-myristate-T4 complexes in which the seven known fatty acid binding sites have been saturated (PDB ID: 1HK5). Here, we have not made use of the latter structural information, because the complexes studied have been obtained under non-physiological conditions (6).

**Figure 4:**
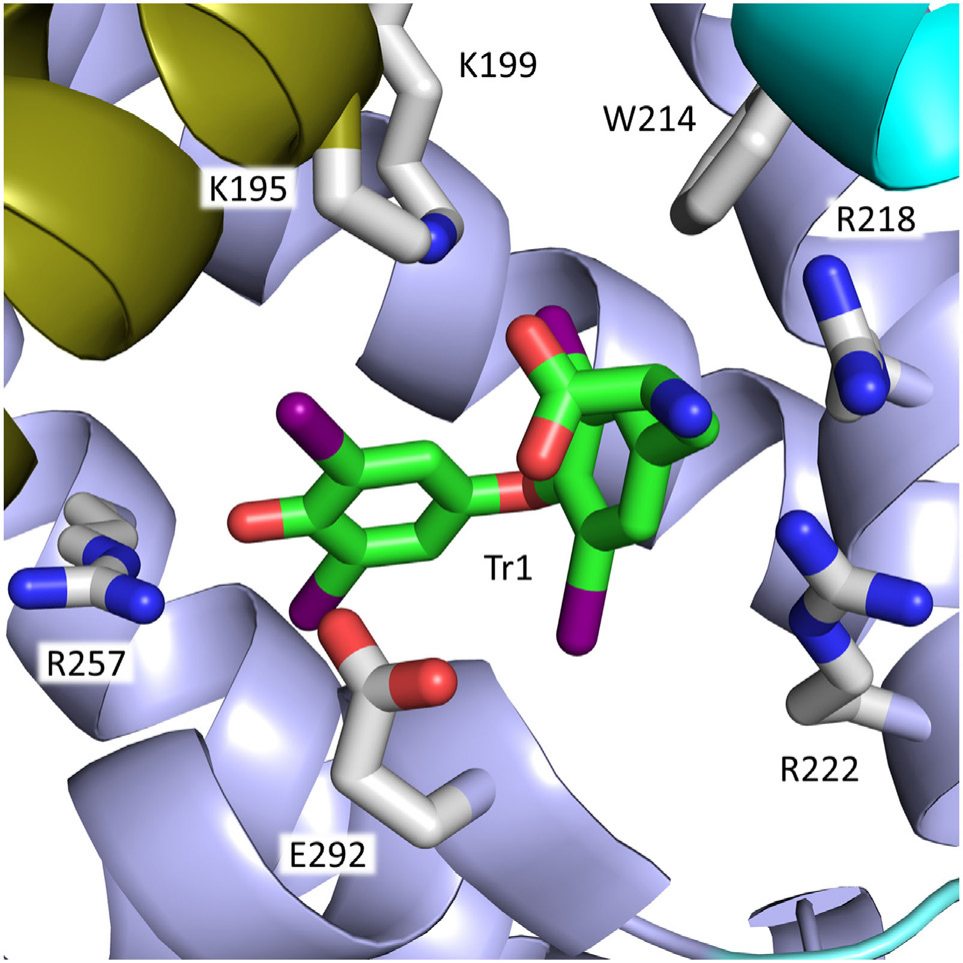
Binding of the cisoid form of T4 to site Tr1 in subdomain IIA of rHSA. Arg218 and Arg222 and other residues in the mature protein of importance for T4 binding are depicted. The color code for T4 in this figure and in Figure 5 is carbon, green; nitrogen, blue; oxygen, red; iodine, magenta. The color code for the subdomains is the same as that used in Figure 3.

The most interesting site in the present context is site Tr1, because it involves the side chains of Arg218 and Arg222 in the mature, native protein (Figure 4). The site is placed in a pocket in subdomain IIA, and T4 binding is mainly the result of hydrophobic interactions between the major part of the hormone and the predominantly hydrophobic pocket. Other forces, which stabilize binding are hydrogen bond interactions between the phenolic hydroxyl of T4 (Figure 1) and the side chains of Tyr150 and Arg257, a salt-bridge interaction between the carboxyl moiety and Lys199 and perhaps also Lys195, and the iodine atoms making hydrophilic contacts with side chains and main-chain carbonyl oxygens within the site (6) (Figure 4). In addition, molecular modeling performed on relaxed protein structure shows a strong interaction between the amino group of T4 and Glu292 (4). T4 binds to the site in a cisoid conformation with the amino-propionic acid and the outer phenolic ring both on the same side of the inner ring of the molecule (6) (Figure 4). This conformation is the result of steric constraints caused by the residues of Trp214, Arg218, and Arg222 (6) (right side in Figure 4), and it is in very good agreement with the conformation determined by molecular docking simulations (4).

Substituting Arg218 or Arg222 for a smaller amino acid increases the size of the binding site and diminishes the steric constraints on bound T4. These changes, and the accompanying, moderate conformational changes of both protein and hormone, result in a stronger ligand binding and can explain the increased T4 binding to R218H (50) and R222I (6, 45). In the case of R218P, however, the moderate conformational changes are combined with a concomitant distortion of the helix main chain (see Figure 4), which allows for a translation of T4 toward the mutated residue. This closer contact results in a very strong T4-binding (6). The affinity of T4 for R218S is intermediate to those of R218H and R218P. R218S has not been studied by X-ray crystallography but modeling data propose that the reason why R218S binds the hormone stronger than R218H is that the hydroxyl of Ser218 is within hydrogen bonding distance to the backbone carboxyl of Trp214 (44).

T4 binding has also been studied by using site-directed mutagenesis. For example, substituting Arg218 for a histidine (4, 38, 50), proline (38), methionine (50), glutamate (51), or an alanine (4, 51) results in all cases in increased T4 binding, and it seems like the smaller the amino acid residue the stronger the T4 binding. In the case of R218A, the binding constant has been reported to increase by as much as two orders of magnitude (51). For Arg222, changing the arginine to the smaller methionine (50), glutamate (51), or alanine (4) also increased binding. Thus, the effect of substituting Arg218 and Arg222 seems to be straightforward and in full accordance with the information from X-ray crystallography. By contrast, the effect of mutating Trp214 seems less evident, because W214L (50) and W214E (51) bind T4 stronger than rHSA, whereas W214A (4) has lesser affinity for the hormone. These findings could indicate that an amino acid of a certain size in position 214 is necessary for T4 binding.

**Figure 5:**
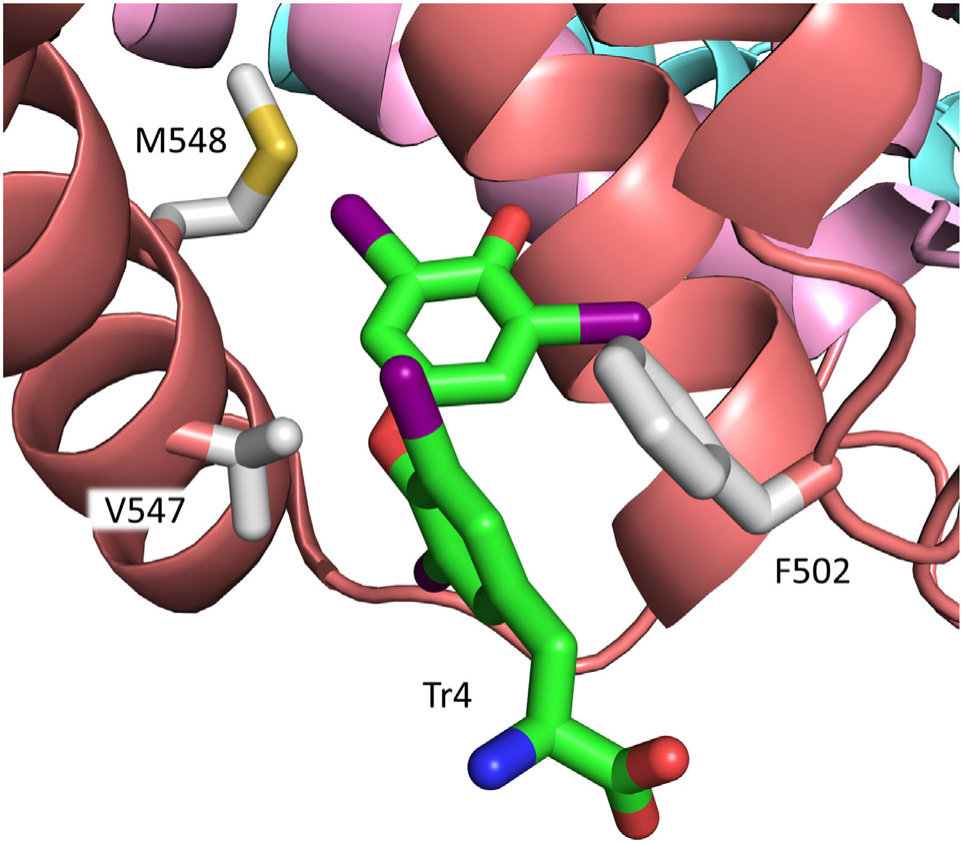
Binding of the transoid form of T4 to site Tr4 in subdomain IIIB of rHSA. Residues of relevance for T4 binding in the mature protein (6) are depicted. The color codes for T4 and for the subdomains are the same as those used in Figures 4 and 3, respectively.

It is widely assumed that Tr1 is the high-affinity binding site for T4 in HSA. However, recent studies strongly propose that this is not the case. As mentioned in section “Other Mutations,” binding experiments with a large series of genetic variants of HSA suggest that the primary site for T4 is situated in subdomain IIIB in the native protein (4). Such a location is in accordance with the results of displacement studies with several marker ligands (4). Thus, the two types of studies indicate that the high-affinity site for T4 is Tr4 and not Tr1 (Figure 3). This new assignment is strongly supported by calculations of MM-PBSA (Molecular Mechanics Poisson–Boltzmann Surface Area) binding energies and by molecular docking performed on relaxed protein structure (4). In contrast with Tr1, Tr4 binds T4 in a transoid conformation, similar to the conformations observed for T3 and T4 bound to the thyroid hormone receptor and TTR (6). Phe502, Val547, and Met548 seem to play an important role for binding of T4 to Tr4 (6) (Figure 5). In conclusion, Tr4 is the primary T4 binding site in native HSA, whereas Tr1 is a secondary binding site, which is mutated to a high-affinity site in FDH-T4 albumin (4). Placing the high-affinity binding site (Tr4) and the one which can result in FDH-T4 (Tr1) in two very different parts of HSA is not trivial. The different locations are of clinical relevance, because persons with and without the syndrome can have different types of interactions, and thereby complications, when given albumin-binding drugs. Furthermore, the molecular information is useful when designing drugs based on T4 analogs.

## Displacement of T4 by Other Ligands Simultaneously Binding to FDH-T4 Albumin

T4 binding to FDH-T4 albumin can be modified by simultaneous co-binding of another ligand. For example, chloride ions can displace T4 bound to the protein. This effect has analytical consequences, because determination of free T4 in serum from FDH-T4 individuals will be too high when using chloride ion-containing buffers (52). In addition, especially individuals with R218P are susceptible to drug-induced thyrotoxicosis if given warfarin, aspirin, or furosemide, which all bind to subdomain IIA of HSA, because they can displace the hormone (38).

## Binding of Other Ligands to FDH-T4 Albumin

In addition to T4, the FDH-T4 causing mutations can influence binding of other ligands, both endogenous and exogenous ones. Thus, fatty acids like octanoate, decanoate, laurate, and myristate bind to the R218H mutant protein with reduced high affinity (53). Also binding of drugs can be modified. For example, high-affinity binding of the widely used warfarin to R218H (54, 55) and R218P (54) is decreased. Binding affinity is reduced to such an extent that warfarin pharmacokinetics may be altered (54). This effect could as well be present for other drugs strongly bound to subdomain IIA of HSA.

## Potential Therapeutic Effects of FDH-T4 Albumin

Acute hyperthyroidism such as thyroid crisis or storm, possibly caused by serine protease cleavage of TBG (6), has to be treated aggressively. In such situations could administration of a strong binder of T4, such as R218A, be useful, because binding will cause inactivation of the hormone. If relevant, administration of a strong T4-binder can also decrease placental transfer of the hormone.

Hyperthyroidism and thyrotoxicosis, complicated by T4-induced liver dysfunction, can be treated with an extracorporeal blood detoxification method such as the molecular adsorbent recirculating system (56). This system combines albumin dialysis with conventional hemodialysis to remove water with soluble toxins and albumin-bound compounds such as T4. The speed with which surplus T4 can be removed would be increased by using a strong T4-binder in the albumin dialysis step.

## FDH-T3 Causing Mutation

### L66P

FDH-T3 is another autosomal dominantly inherited condition affecting thyroid hormone binding to albumin. In this syndrome, the single point mutation has taken place in exon 3 of *ALB*. The normal codon 66 (*CTT*) has been mutated to *CCT*, resulting in the replacement of the normal leucine by proline in the mature protein (Table 1). When measured by radioimmunoassay, the albumin modification results in a high serum concentration of total T3, but a total T4 within the normal range (Table 2). This finding is in contrast to the FDH-T4 cases in which total T4 is always increased (Tables 1 and 2). Both free T3 and free T4 are normal, and all affected persons are clinically euthyroid (46). Binding experiments with diluted serum from affected subjects indicated that the binding affinities for T3 and T4 are increased 40- and 1.5-fold, respectively, as compared to that of unaffected relatives (46).

Although no DNA or protein sequencing was performed, reports suggest the existence of FDH-T3 in Japan. For example, a patient with Graves’ disease was found to have an albumin with markedly enhanced affinity for T3, a slightly increased affinity for T4 and normal binding of rT3 (57). More recently, three sisters were reported to have high serum levels of T3 but not of T4 or rT3 (58).

The prevalence of this anomaly is unknown, but the syndrome must be rare, because only a few reports exist on the subject. This point of view is supported by the fact that L66P is not mentioned in the Exome Aggregation Consortium Website (47). According to the website, it means that the frequency of the mutant albumin is less than 0.83 in 100,000.

### L66V

The Exome Aggregation Consortium Website (47) reports on the existence of a L66V isoform of HSA caused by a missense mutation of the corresponding codon from *CTT* to *GTT*. This variant was found in two individuals of European (Finnish) origin with a frequency of 1.7 in 100,000. However, it is neither present in the Albumin Website (16), nor has it been reported to cause FDH-T3, at least according to our knowledge.

## Structure of T3-Binding Site in HSA

**Figure 6:**
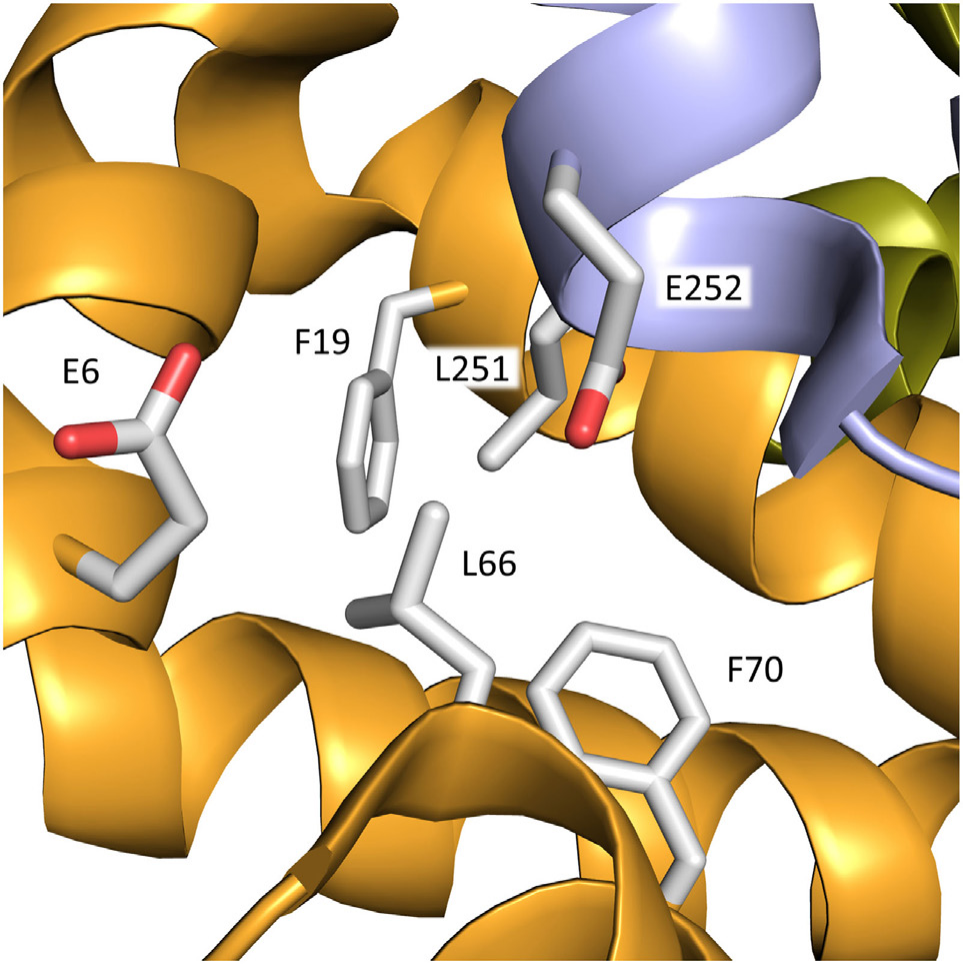
Binding site for T3 in subdomain IA of rHSA. The mutation L66P increases the affinity for the hormone. In addition to Leu66, bulky residues within a distance of 3–5 Å from the residue in the mature protein are depicted. The color code for the subdomains is the same as that used in Figure 3.

In contrast to binding of T4, not much is known about the number and location of the T3-binding site(s). T3 likely binds to a site in subdomain IA in which Leu66 is an important element (Figure 6), and substitution of proline for the leucine increases the affinity for the hormone. As seen in Figure 6, Leu66 is surrounded by several bulky residues. Therefore, the molecular explanation for the stronger T3 binding could be principally the same as that found for FDH-T4; i.e., the mutation diminishes the steric constraints on bound T3. Alternatively, or additionally, proline could cause conformational changes in the helix, which would allow for closer contact between hormone and protein.

## Conclusion

Familial dysalbuminemic hyperthyroxinemia and FDH-T3 are dominantly inherited conditions caused by genetic variants of HSA with increased affinity for thyroid hormones. FDH-T4 is caused by mutation of Arg218 or Arg222, whereas FDH-T3 is due to substitution of Leu66. The syndromes, especially FDH-T4, can be fairly common in certain populations. The persons affected are euthyroid, because the concentrations of free hormones are within the normal range, and thus do not need treatment. However, many methods for the determination of free hormone give erroneously high values. These laboratory results have often led to unnecessary medical and surgical treatment. Many molecular details are known about binding of T4, but not of T3, to normal HSA and to FDH-T4 causing mutants. The latter mutations can also affect binding of other ligands and can most probably cause modified pharmacokinetics of albumin-binding drugs.

## Author Contributions

UK-H, MG, and LM made the literature research and wrote the manuscript. All the authors have read and approved the manuscript.

## Conflict of Interest Statement

The authors declare that the research was conducted in the absence of any commercial or financial relationships that could be construed as a potential conflict of interest.
